# Psychiatric Care for Children and Adolescents After the Onset of Type 1 Diabetes: Implementation of a Multidisciplinary Programme

**DOI:** 10.1192/j.eurpsy.2025.847

**Published:** 2025-08-26

**Authors:** J. Sánchez-Cerezo, A. Guzmán De Lazaro, L. García Murillo, R. Paricio Del Castillo, A. Cañuelo Márquez, A. Pascual Aranda, I. Palanca Maresca

**Affiliations:** 1 Department of Psychiatry, Division of Child and Adolescent Psychiatry, Puerta de Hierro University Hospital, Majadahonda, Madrid, Spain

## Abstract

**Introduction:**

Type 1 Diabetes Mellitus (T1DM) is a chronic autoimmune disease characterized by the destruction of insulin-producing beta cells in the pancreas. It commonly presents in childhood or adolescence. As one of the most prevalent chronic conditions in children, T1DM significantly affects the lives of patients and their families. In Western Europe, the incidence rate is 10-20 new cases per 100,000 individuals per year. T1DM requires structured insulin self-management, blood glucose monitoring, physical activity, and a healthy diet what can be challenging for children and adolescents. T1DM in children and adolescents is associated with various psychosocial problems, including a heightened risk of psychiatric disorders such as depression or anxiety, and problems with eating, alcohol use, or sleep. Effective management requires a multidisciplinary approach, involving psychiatric care to address mental health concerns and improve disease management.

**Objectives:**

Here we present a novel programme that aims to address the integration of psychiatric care for children and adolescents with T1DM by facilitating early detection of mental health issues, enhancing education for patients, families, and healthcare providers, promoting multidisciplinary collaboration, and offering tailored treatment. We present preliminary outcomes of our programme.

**Methods:**

Our programme at the Puerta de Hierro University Hospital involves comprehensive mental health assessments for paediatric T1DM patients. Paediatricians refer patients to Child Psychiatry after T1DM onset. The initial evaluation includes a clinical history, mental health background, risk factor assessment, and the completion of routine measures. Follow-up is scheduled based on the clinical presentation with a maximum treatment time of 12 months. Image 1 shows the flowchart with the referral and treatment process in our programme. Our multidisciplinary team consists of one child and adolescent psychiatrist, one clinical psychologist and one mental health nurse and we work collaboratively with paediatricians.

**Results:**

From October 2023 to September 2024, we received 13 referrals (61.5% females) with a mean age of 10.4 years. Of those, 38.5% had a diagnosis of emotional disorder, 30.8% attention-deficit hyperactivity disorder, 7.7% conduct disorder, 15.4% required parenting support, and 15.4% required psychological support. Patients and families show high rates of improvement regarding their mental health problems and psychological adjustment and are satisfied with the care provided.

**Image 1:**

**Figure:**
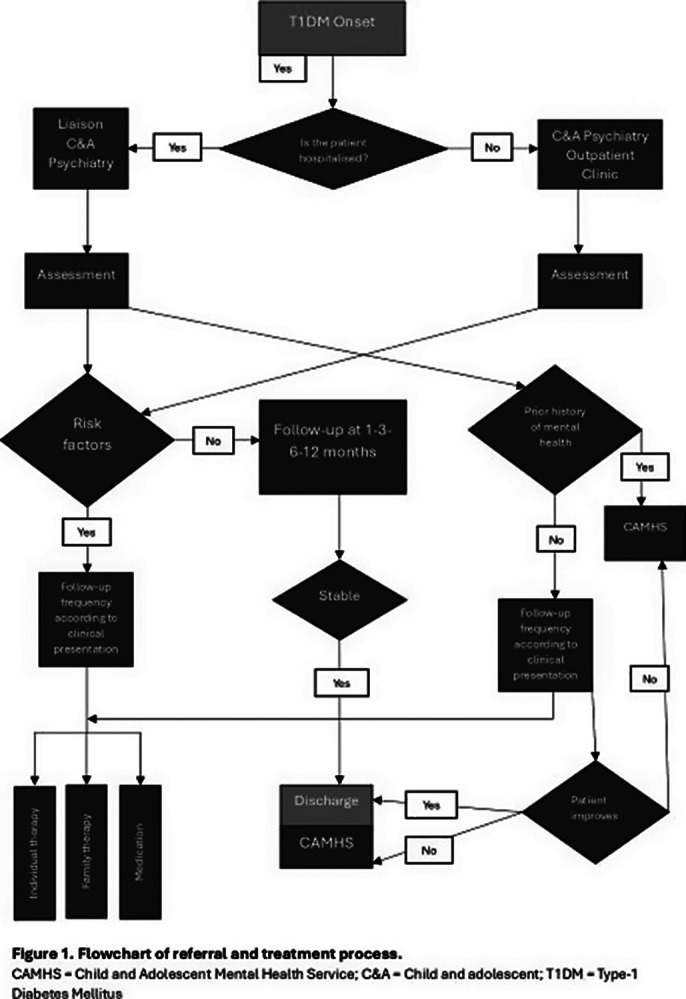

**Conclusions:**

Early and effective psychiatric intervention is crucial in managing the complex needs of children and adolescents with T1DM. The implementation of this multidisciplinary programme is feasible, and it has shown promising results. In the future, a randomised controlled trial should be conducted to assess the effectiveness of this intervention.

**Disclosure of Interest:**

None Declared

